# Introduction of *West Nile Virus* in the Middle East by Migrating White Storks

**DOI:** 10.3201/eid0804.010217

**Published:** 2002-04

**Authors:** Mertyn Malkinson, Caroline Banet, Yoram Weisman, Shimon Pokamunski, Roni King, Marie-Th, r, se Drouet, Vincent Deubel

**Affiliations:** * Kimron Veterinary Institute, Beit Dagan, Israel; † Veterinary Services and Animal Health, Beit Dagan, Israel; ‡ Nature and Parks Authority, Jerusalem, Israel; § Pasteur Institute, Lyon, France

**Keywords:** *West Nile virus*, white storks, domestic geese, genomic sequences, and bird migration

## Abstract

*West Nile virus* (WNV) was isolated in a flock of 1,200 migrating white storks that landed in Eilat, a town in southern Israel, on August 26, 1998. Strong, hot westerly winds had forced the storks to fly under considerable physical stress before reaching the agricultural land surrounding the town. Most of the flock were fledglings, <1 year old, which had hatched in Europe. Thirteen dead or dying storks were collected 2 days after arrival and submitted to the laboratory for examination. Four WNV isolates were obtained from their brains. Out of 11 storks tested six days after arrival, three had WNV-neutralizing antibodies. Comparative analysis of full-length genomic sequences of a stork isolate and a 1999 flamingo isolate from the USA showed 28 nucleotide (nt) (0.25%) and 10 amino acid (0.3%) changes. Sequence analysis of the envelope gene of the stork isolate showed almost complete identity with isolates from Israeli domestic geese in 1998 and 1999 and from a nonmigrating, white-eyed gull in 1999. Since these storks were migrating southwards for the first time and had not flown over Israel, we assume that they had become infected with WNV at some point along their route of migration in Europe.

Since the early 1950s, West Nile fever (WNF) epidemics in human populations of many African, Middle Eastern, and some Mediterranean countries have occurred at approximately 10-year intervals. With the exception of a minor outbreak in France in 1962 (*1*), however, WNF was considered unimportant to human health in Europe. The situation changed radically in 1996, when an epidemic swept through the city of Bucharest, Romania; nearly 400 human cases of encephalitis occurred and approximately 40 people died (*2*). Several more cases were reported in 1997 and 1998 (*3*). Then, July through September 1999, a widespread epidemic of WNF was reported in southern Russia involving approximately a 1,000 cases with at least 40 dead (*4*). Within the 5-year period from 1996 through 2000, WNF was also diagnosed in isolated human patients in the Czech Republic, in 14 horses in Tuscany, Italy, and in 78 horses from the Herault and Gard Provinces, France (*5*–*7*). Another European report of a WNF epidemic was received from West Georgia (former Soviet Union) in 1998 (*8*). In July through October 1999, an outbreak of WNF in humans, horses, and wild and zoo birds was reported in New York (*9*) and neighboring states. This outbreak was caused by a virus almost identical genetically to one isolated previously from domestic geese in Israel in 1998 (*10*).

WNF epidemics occur in the late summer and early fall months in temperate regions of Europe when bird migration is at its peak and mosquito populations are greatest. Nevertheless, WNV has been isolated occasionally from actively migrating birds in Europe, emphasizing their importance as carriers of several arboviruses (*11*). On the other hand, WNV antibodies have been found in wild birds caught in many countries of Europe, Africa, and Asia (*12*).

The white stork (*Ciconia ciconia*) migrates over the Middle East each fall in numbers estimated at 500,000, (*13*) but for reasons connected with difficulties in catching them, none of the published serosurveys of wild birds from Europe, Africa, and Asia mention storks. In early September 1998, we received dead storks and serum samples from the town of Eilat in southern Israel. The storks and samples were from a flock of 1,200 birds that had landed in Eilat on August 26, 1998. The storks were weak, having been blown off their usual route of migration through Jordan. The storks’ appearance in Eilat was a very rare sight because they normally fly in thermals which take them on a route down the Arava stretch of the Syrio-African Rift Valley where they turn southwest south of the Dead Sea and cross the Sinai Peninsula into Africa (*14*). The previous recorded sighting of storks in Eilat had been in August and September 1980 (R. Yosef, pers. comm.).

In 1998, unusually strong winds had carried them eastwards, and in an attempt to reach the Sinai, the flock had resorted to powered (flapping) flight. From their bodily conformation, bright golden beaks and legs and wing feathering, most of the flock was classified as juveniles, i.e., hatched in 1998. In this report, we present the results of virologic and serologic studies on this flock and compare the sequences of the stork WNV genome with Israeli isolates from geese (*Anser anser domesticus*) in 1998 and 1999 and a White-eyed Gull (*Larus leucophthalmus*) in 1999. In addition, we include information on the serology of migrants and resident storks in Israel from which blood samples were taken in 1998 through 2000. Based on these data and an evaluation of the routes and seasons of the storks’ migration, we suggest that WNV could have been imported into Israel by storks that were infected along their fall migratory routes over southeast Europe and the Near East.

## Materials and Methods

### Storks

Dead and dying storks were collected from the area around the landing site 2 days after arrival of the flock on August 26. They were immediately placed in cold storage until transported to the laboratory on September 4, September 9, and October 8, when the birds were thawed and weighed, and necropsies were performed. A total of 13 frozen storks were received.

### White-eyed gulls

A small breeding colony of approximately 20 gulls was housed in a closed pen with wire-mesh walls at the Department of Zoology, University of Tel Aviv. In November 1999, several birds were found paralyzed, and two had died.

### Isolation of virus

Brains were removed aseptically from the storks and gulls and homogenized by grinding in borate-buffered saline (pH=7.6). The homogenate was centrifuged at 1,500 rpm for 10 min. Other tissues were not examined. The supernatant was removed and used for intracranial inoculation of suckling mice and infection of Vero cells and yolk sac inoculation of 7-day-old embryonated eggs. Embryonic mortality occurred 3 to 4 days after inoculation and the embryos were colored cherry red but without hemagglutinating activity for chicken erythrocytes in the allantoic fluids. An arbovirus was therefore suspected. Likewise, morbidity of the mice was accompanied by spastic paralysis, a further sign of virally induced encephalitis. In Vero cell culture, a cytopathic effect (CPE) was seen within 3 to 5 days of inoculation.

### Serology

 Storks were aged either as fledglings (<1 year old) or mature birds by their wing feathering and intensity of the yellow pigment of the beaks and legs (E. Gorni, pers. comm..). Blood samples were taken from the Eilat flock 6 days after arrival. Other storks from this flock were placed in a nature reserve to recuperate, and blood samples were taken in September and October. In October 1998 and September 1999, storks were caught as they were migrating over Israel, and blood samples were taken. The January 2000 flock consisted of late migrators that were caught while roosting. The blood samples were taken from the White-eyed Gulls 1 week after isolation of WNV.

Sera were diluted twofold commencing at 1:10 in WNV suspension containing 100 50% tissue culture infective dose (TCID_50_)/0.1 mL in microwell plates. The virus-serum mixture was allowed to stand at room temperature for 1 hr, and a suspension of 10^5^ Vero cells in 0.1 mL was added to each well. The plates were incubated at 37^°^C in 5% CO_2_/95% air for 4 days. The neutralizing titer of the serum was calculated as the highest dilution with 50% CPE.

### Indirect Immunofluorescence Assay (IFA)

 When CPE of the infected Vero cell culture was observed, a glass coverslip was placed in the dish receiving the succeeding passage. Three days later the coverslip was removed and the cells fixed with cold acetone. Identification of the cytopathic agent was performed by indirect immunofluorescence. Panels of monoclonal antibodies to *Alphavirus*, and Flaviviruses (Table 1) were reacted with the Vero cells and then incubated with flourescein isothiocyanate (FITC)-labeled anti-mouse immunoglobulins. The *Flavivirus* monoclonal antibodies included 2B4, 6B8, and 6E12 from South Africa; 5F10, 1C9, and 1B4 from Israel, F7/101 from Oxford, U.K.; and 3H6 and 813 from Townsville, Australia. The *Alphavirus* (*Sindbis virus* [SINV]) antibodies were 30.11 and 30.12 from Oxford and 2F2 from Australia. Counter-staining was performed with Evans blue.

**Table 1:** Monoclonal antibodies used characterization of goose, stork, and gull isolates study, Israel, 1998 and 1999

**MAb**	**Reactivity**	**Reference**
2B4	WNV-type specific (E gene)	*15*
6B8	WNV-type specific (E gene)	*15*
6E12	WNV-type specific except KUN (NS4a gene)	*15*
5F10	WNV specific	Bat-El Lachmi
1C9	Flaviviruses	Bat-El Lachmi
1B4	Flaviviruses	Bat-El Lachmi
F7/101	West Nile	*16*
6D12	Flaviviruses except *Edge Hill* and *Dengue virus*	*17*
3H6	*Murray Valley encephalitis virus* (*Flavivirus*)	*17*
813	*Yellow fever virus* (*Flavivirus*)	*18*
30.11	SINV	*19*
30.12	SINV	*19*
2F2	SINV	^a^

WNV=*West Nile virus*; SINV=*Sindbis virus*. ^a^TropBio, Townsville

### RT-PCR

Brain extracts were performed as described above and the supernatant used to extract RNA with the QIAamp Viral RNA kit (Qiagen, Valencia, CA) and the RNAs resuspended in 60 μL of Viral Lysis Buffer (Qiagen, Valencia, CA) elution buffer according to the manufacturer’s protocol. Reverse transcription-polmenase chain reaction (RT-PCR) was performed on a gene fragment of the envelope protein using the primer pair WN132 (5′ GAAAACATCAAGTATGAGG 3′) and WN240 (5′ GAGGTTCTTCAAACTCCAT 3′) (genome positions 1,402 and 1,656), resulting in the synthesis of a 255-bp product (*20*). The resulting DNA fragment was visualized on 1.5% agarose gel stained with ethidium bromide.

### Gene Sequence Analysis

WNV RNA was extracted from the second passage of Vero cell-infected culture fluid after isolation in a suckling mouse brain, using the QIAamp Viral RNA kit (Qiagen). Viral RNA was extracted from supernatant fluid according to the manufacturer’s protocol and the RNA resuspended in a final volume of 100 μLof RNase-free water. Six overlapping double-stranded cDNA templates were generated by using WNV-specific primer pairs provided by the Centers for Disease Control and Prevention (*9*). Additional primers with sequences designed from the sequence of WN-NY99 were used to amplify about 100 nucleotides from the far 3′ and 5′ extremities (Table 2). The envelope protein genes from WN viruses isolated in 1998 and 1999 from geese and in 1999 from a White-eyed Gull were amplified using primer pairs designed in the corresponding strands.

**Table 2:** Oligonucleotide primers used for framing the entire *West Nile virus* (WNV)^a^ genome and the envelope (E) gene, Israel 98—ST1

Localization in the WNV genome	N° nucleotide at the 5′ end	Sequence
5′ -end	1	AGTAGTTCGCCTGTGTGAGCTGACAAAC
3′ -end	10,934 (c)	AGATCCTGTGTTCTCGCACCACCAGCCAC
M-gene	889	GGATGGATGCTWGGKAGCAAC
NS1-gene	2,557 (c)	CCATCCAAGCCTCCACATC

(c)=complementary sequence; W=(A+T); K=(G+T).

Amplified cDNAs were purified by ion-exchange chromatography and precipitated with 2v of isopropanol. Both strands of the purified cDNAs were sequenced by using the Taq Dye Deoxy Terminator Cycle sequencing kit (Perkin Elmer Corp./Applied Biosystem, Norwalk, CN) using primers spaced about 400 bases apart on the genome (*9*). Cycle sequencing was performed by combining about 0.2 pmols of purified cDNA and 30 pmols of primer and following the manufacturer’s protocol. Sequences were aligned with the Clustal W alignment (*21*).

## Results

### Virus isolation from Storks and Gulls.

A total of four Flaviviruses were isolated from storks in Vero cells—one from a pool of three brains from the first group of dead storks collected on August 28, 1998; one isolate from one of six individual brains; and two isolates from four brains also collected on August 28. The three pooled stork brains from the first group were also injected into three litters of baby mice, causing paralysis. As noted previously, all the storks had been collected 2 days after landing and were frozen immediately. The four isolates were confirmed as WNV by indirect immunofluorescent antibody (IFA) using monoclonal antibodies and by RT-PCR. No *Alphavirus* reactivity was detected in any of the brains by IFA (Table 3). One WNV was isolated from the brain of one of the White-eyed Gulls found dead in November 1999 in Tel Aviv (data not shown).

**Table 3:** Summary of viral isolations and polymerese chain reaction (PCR) examinations of the 13 storks that arrived in Eilat, Israel, August 26, 1998

Date of collection (1998)	Date of laboratory examination	No. of storks examined	No. of WNV isolates (Vero cells)	No. of brains PCR positive
August 28	Sept 4	3^a^	1 ^a^	3 ^b^
August 28	Sept 9	6	1	2
August 28	Oct 8	4	2	4
Total		13	4	9

^a^ Pooled sample. ^b^ From three litters of suckling mice inoculated with the pool of three stork brains. WNV=*West Nile virus*.

### RT-PCR

All three homogenates of the mouse brains inoculated with the brains of the first group of storks were RT-PCR positive. Two of the six brains of the second group and all four of the third group were RT-PCR positive. Thus, a total of one pool of 3 brains and 6 individual brains from 10 storks tested were RT-PCR positive (Table 3).

### Sequence Analysis

The full-length RNA genome of the WNV isolate from one of the stork-1998 (IS98-ST1) has been sequenced. With the exception of about 30 nucleotides at each end of the genome used as primers for cDNA amplification (Table 2), the complete nucleotide sequence of the viral RNA has been determined (data not shown). The WNV genome arrangement is similar to those published for WNV-Nigeria, WNV-NY99, and HNY1999 (*9*,*22*,*23*). The genome is 11,029 nucleotides in length and contains one long open reading frame of 10,302 nt starting at nt 97. Nucleotide sequence comparison of IS98-ST1 with WNV-NY99 (*9*) showed 28 mutations in the coding region (99.75% similarity). Of the mutations, five were transversions, and the rest occurred at the third codon. Ten amino acid changes were found. The change at position 51 (A51 to V) in WNV-NY99 E protein was not found in the HNY1999 sequence. Other mutations were observed in NS1 (N17 to S), NS2A (R165 to G), NS2B (G82 to D and E83 to G), NS3 (P496 to L and E521 to D), and NS5 (S54 to P, N280 to K, and A372 to V).

The envelope gene sequence of IS98-ST1 (GenBank accession No. AF 481864) was compared to those from goose98 (GenBank accession No. AY033388) and goose99 (GenBank accession No. AY033391) and gull99 (GenBank accession No. AY033390) and included HNY 99 as the consensus sequence (Figure). The comparison showed 3 nt changes, of which one was unique for IS98-ST1 strain (position 1,179), one common to goose99 and gull99 (position 729) and one shared by gull99 and IS98-ST1 (position 3). The 501 amino acids of the E genes of the four strains (IS98-ST1, goose98, goose99, and gull99) were identical (data not shown).

**Figure:**
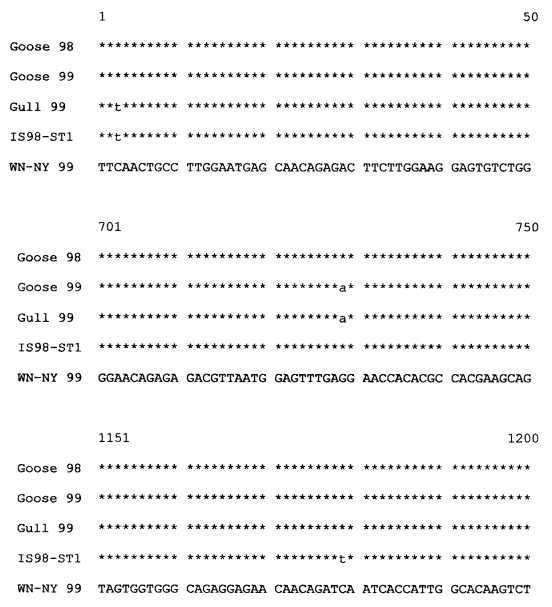
Abbreviated alignment of E gene sequences of Israeli isolates Goose 98, Goose 99, White-eyed gull 99, and IS98-ST1 with the consensus sequence of WN-NY99. The nucleotide numbers correspond to their location in the envelope gene.

### Serology

Table 4 presents the WNV neutralization titers of groups of storks according to the month and year of the blood samples, the storks’ age, and geographic location. In 1998 sera were collected from the Eilat flock on September 2, and from eight older storks in a wildlife sanctuary during September and October. Late migrating storks were also captured in northern Israel during their autumn migration in October, and 10 birds from various bird sanctuaries were also sampled. Between January and May 1999, all five storks resident in northern Israel had WNV antibodies. With the fall migration, blood samples were taken from a group of six storks, consisting of four fledglings and two older birds. One stork in each age group had WNV antibodies.

**Table 4:** *West Nile virus* neutralizing antibodies of storks caught at various sites, Israel, 1998–2000

Date of collection		Location	Age	No. pos/ No. test	SN^a^ titer
Year	Month				
1998	Sept 1	Eilat I	<1 year	3/11	2x40, 1x>1280
	Sept 9	Eilat II	Adult	3/3	320, 640, 1280
	Oct 8	Eilat III	Adult	5/6	20->1280
	Oct 12	Kfar Rupin (migrating)	Adult	4/8	2x80, 2x640
	Oct 20	N. Israel (Resident)	Adult	6/10	1x40, 4x80,1x160
1999	Jan 10	N. Israel	Adult	2/2	80, 160
	Apr 10	(Resident)		1/1	80
	May 12			2/2	160, 320
	Sept 5	Kfar Rupin	<1 year	1/4	40
		(migrating)	Adult	1/2	80
2000	Jan 3	Neve Eitan (migrating)	Adult	9/12	4x80, 4x160, 1x320
	Jun 15	Golan Heights (resident breeders)	3 months	0/4	<10
		Total	<1 year	4/19	(21.1%)
			Adults	33/46	(71.7%)
				37/65	(56.9%)

^a^SN=serum neutralization

Storks examined through June 30, 2000, consisted of two groups, one was a flock of overwintering adults that had migrated in September 1999 and the others were four fledglings hatched in April 2000 from parents that had overwintered and bred on the Golan Heights. Antibodies were detected in 9 of the 12 adults but in none of the young birds.

Thus, a total of 65 stork sera were examined of which 19 were from birds <1 year old. In the 19 birds <1 year, neutralizing antibodies were found in 4 of them (21.1%), whereas in the older group of 46 storks, 33 (71.7%) were seropositive.

Of the 11 White-eyed Gull sera examined 1 week after the isolation of WNV, 8 were seropositive, 6 had titers > 1:1280, one of 1:640, and one of 1:80, and three were < 1:10.

## Discussion

In this report we describe the isolation of WNV and detection of virus activity by RT-PCR in the brains of storks that were grounded while migrating southward on a route that took them along the eastern edge of the Syrian-African Rift Valley, Jordan. Strong winds in the last days of August 1998 carried the flock off course, but by active flying the birds had landed in Eilat. The relatively high number of isolates and PCR-positive brains may be due to a compounded effect of the stress imposed on the flock by migration and the strenuous physical efforts exerted by the birds in flying back on course (*24*,*25*). Recoveries of SINV and WNV from white storks caught in southern Sinai during the fall of 1998 through spring 1999 as they migrated towards Africa have been reported recently (*26*). Viruses were isolated from 13 of 25 birds; of these 12 were SINV and one was WNV.

We were able to gauge the age of the serum samples according to whether the stork had hatched in the year it was caught (fledgling bird) or in a previous year. With very few exceptions all the fledglings, and notably the Eilat flock, had hatched in Europe in the spring of the same year and had yet to complete a full migratory cycle by overwintering in Africa. Thus, these birds could have been exposed to WVN in Europe, either near the nesting sites or along the route of migration, especially along the River Danube and its tributaries. The ornithologic and epidemiologic features of the Danube Delta have recently been described by Savage et al. (*27*) in relation to the 1996 epidemic in Bucharest.

The stork summer migration from northern European countries follows the Danube to reach the Black Sea and is in the reverse direction to the storks’ breeding sites in central and northern Europe during the spring migration (*14*). Since none of the fledglings were leg-banded, we cannot identify where infection could likely have occurred. It is unlikely that infection occurred in Eilat because the injured and dead birds were collected within 2 days of landing there. In contrast, seropositive older birds were most likely exposed either to a disease-endemic environment, as is found in most African countries where they overwinter (*28*), or in Europe, where they breed. Savage et al. (*27*) has recently shown that serum samples from domestic and wild fowl collected in the vicinity of Bucharest during the 1996 WNV epidemic had neutralizing antibody to WNV. Thirty (41%) of 73 domestic birds, including 13 ducks and 1 goose, were positive, although the actual titers were not stated.

With the appearance of the flock in Eilat in August, the fall migration was proceeding over Israel at the same time. In September a WNV outbreak affecting young geese was recorded on farms throughout Israel. Mortality was as high as 40% in some flocks (*10*). The marked susceptibility of domestic geese to WNV had not been reported previously in Israel or elsewhere, although goose management had not changed over the years with flocks traditionally reared in open farmyards. The emergence of the goose as an incidental host therefore appears to be related to a change in the WNV genome possibly related to what had occurred in Bucharest in 1996. A strong homology in the nucleotide sequence was observed between the goose-1998/1999, gull-1999, and stork-1998 isolates. Comparative analysis of a short E gene fragment of several WNV strains from Romania 96, Kenya 98, and Russia 99, has yielded a high level of identity (96%) among the strains (*9*,*28*–*30*). This suggests that the WNV epizootic observed in Israel between 1997 and 2000 may be attributable to viruses that were circulating in eastern Europe or elsewhere in the Near East since 1996. On the other hand, the identification in 2000 of a second genotype circulating in Israeli human and avian populations clouds the molecular epidemiologic picture (*31*, Banet et al., manuscript in preparation). Based on a comparison of a 1,278-bp sequence of the E gene and a 1,648-bp fragment spanning the preM, M, and the 5′ terminus of the E gene, this second genotype resembled even more closely (99% homology) the Romanian 96, Kenyan 98, and Volgograd 99 strains. Therefore, a different migrating flock or species might have introduced this variant.

The observation that IS98-ST1 is also highly virulent for zoo and wild birds is mirrored by reports from the northeastern United States, where many species of wild birds succumbed to infection (*32*,*33*). A unique feature of the Israeli isolate was its marked pathogenicity for flocks of young geese between 3 and 12 weeks of age, causing considerable mortality, whereas the New York isolates targeted mainly the crow (*34*). Despite the phylogenetic similarity of these two isolates, no plausible explanation has been offered for the unique appearance of WNF in North America in 1999. Attempts to link transatlantic migration of birds, mainly from southern and western Europe, with the dispersion of WNV have identified several candidate species that migrate across the Atlantic Ocean (*35*). In this respect the role of water birds, especially members of the gull family, should also be considered as carriers of flaviviruses.

The emergence of the epidemic in Romania in 1996 was a unique epidemiologic event that heralded the renewed spread of WNV in Europe after more than 30 years of silence. The virus’ dispersion through the Near East and beyond follows a pattern that is best explained by bird migration.
